# Gene and Phenotype Expansion of Unexplained Early Infantile Epileptic Encephalopathy

**DOI:** 10.3389/fneur.2021.633637

**Published:** 2021-06-07

**Authors:** Xianyu Liu, Qiyang Shen, Guo Zheng, Hu Guo, Xiaopeng Lu, Xiaoyu Wang, Xiao Yang, Zixuan Cao, Jing Chen

**Affiliations:** ^1^Department of Neurology, Children's Hospital of Nanjing Medical University, Nanjing, China; ^2^Department of Pediatric Surgery, Children's Hospital of Nanjing Medical University, Nanjing, China

**Keywords:** encephalopathy, epilepsy, infantile, gene, phenotype

## Abstract

**Objective:** The genetic aetiology of epileptic encephalopathy (EE) is growing rapidly based on next generation sequencing (NGS) results. In this single-centre study, we aimed to investigate a cohort of Chinese children with early infantile epileptic encephalopathy (EIEE).

**Methods:** NGS was performed on 50 children with unexplained EIEE. The clinical profiles of children with pathogenic variants were characterised and analysed in detail. Conservation analysis and homology modelling were performed to predict the impact of *STXBP1* variant on the protein structure.

**Results:** Pathogenic variants were identified in 17 (34%) of 50 children. Sixteen variants including *STXBP1* (*n* = 2), *CDKL5* (*n* = 2), *PAFAH1B1, SCN1A* (*n* = 9), *SCN2A*, and *KCNQ2* were *de novo*, and one (*PIGN*) was a compound heterozygous variant. The phenotypes of the identified genes were broadened. *PIGN* phenotypic spectrum may include EIEE. The *STXBP1* variants were predicted to affect protein stability.

**Significance:** NGS is a useful diagnostic tool for EIEE and contributes to expanding the EIEE-associated genotypes. Early diagnosis may lead to precise therapeutic interventions and can improve the developmental outcome.

## Introduction

Epileptic encephalopathy (EE) describes a group of severe intractable seizures and neurological disorders in which epileptic activity itself may contribute to severe neurologic and cognitive impairment, beyond the damage that would be expected from the underlying pathology alone, and these effects can worsen over time (1). Early infantile epileptic encephalopathies (EIEEs) occur during infancy and comprise a spectrum of syndromes such as Ohtahara syndrome (OS), West syndrome (WS), epilepsy of infancy with migrating focal seizures, and Dravet syndrome (DS) (2).

In addition to common metabolic and structural abnormalities, genetics play an important role in the aetiology of EIEEs (3). EIEEs exhibit overlap and pleiotropy, which makes it challenging to establish genotype-phenotype correlations. The recent advent and improvement of next generation sequencing (NGS) have provided a significant advancement in the diagnosis of epileptic patients (4). In this study, NGS was performed in a cohort of Chinese children with unexplained EIEEs. We analysed genotype-phenotype correlations and elucidated therapeutic effects and adjustments.

## Methods

### Patients

A total of 50 patients with EIEEs without specific aetiologies in the department of neurology, at the Children's Hospital of Nanjing Medical University in China were recruited between 2015 and 2018. Other aetiologies such as definite perinatal brain injury, infection of the central nervous system, typical inherited metabolic disorders and abnormal detection of chromosomal microarrays were excluded in these patients. Clinical data including age of seizure onset, type and frequency of seizures, video electroencephalography (VEEG) recording, brain magnetic resonance imaging (MRI), response to antiseizure medications (ASMs), and comorbidities were collected. Electro-clinical syndromes were defined and classified according to the International League Against Epilepsy (ILAE) (5). All patients were followed up with by telephone or outpatient visits every 3 months until October 2019. The study protocol was approved by the hospital ethics committee and all parents provided informed consent for their children's participation.

### Next Generation Sequencing

Genomic DNA was extracted from peripheral leukocytes from patients and their parents. Whole-exome sequencing (WES) or epilepsy-associated gene panel analyses with the Illumina NextSeq 500 platform (Illumina, San Diego, CA, USA) were performed at accredited commercial labs. The epilepsy-associated gene panel contained more than 500 genes and continued updating over time. Copy number variations (CNVs) were analysed by copy number variation sequencing (CNV-Seq) based on the NGS platform. Sanger sequencing was used to validate variants and to analyse segregation in pedigrees. Variants were analysed and annotated according to Exome Aggregation Consortium (ExAC), 1,000 Genomes, Clinvar, dbSNP, Online Mendelian Inheritance in Man (OMIM), and the Human Gene Mutation Database (HGMD). Effects of variants on function were predicted by SIFT, PolyPhen-2, and MutationTaster. Variant pathogenicity was evaluated by the American College of Medical Genetics and Genomics (ACMG) scoring system, together with clinical presentation (6).

### Homology Modelling

Multiple sequences including mutated regions from different species were aligned using the Clustal Omega website, to determine whether the variants are located in an evolutionarily conserved domain. We selected the protein crystal structures of 4JEH and 4JEU from Protein Data Bank (PDB; www.rcsb.org) as templates for STXBP1. Molecular structure modelling of mutant and wild-type proteins was achieved using the SWISS-MODEL online server (7) and PyMOL (www.pymol.org).

## Results

Pathogenic variants of 17 genes were identified in 50 patients (34%) and pathogenic CNVs were not detected. Sixteen variants including *STXBP1* (*n* = 2), *CDKL5* (*n* = 2), *PAFAH1B1, SCN1A* (*n* = 9), *SCN2A*, and *KCNQ2* were *de novo* heterozygous variants, and one (*PIGN*) was a compound heterozygous variant; 11 out of 17 were novel variants. Five patients underwent WES and 12 patients had epilepsy-associated gene panel testing. The male to female ratio was 9:8. The mean seizure onset was 4.5 months (range: 8 days to 11 months). All variants are predicted to be “Pathogenic” according to ACMG standards. The variants and clinical features of the patients are listed in Table 1. Multiple sequence alignments and comparative protein structures of two *STXBP1* missense variants are shown in Supplementary Figures 1, 2.

**Table 1:** Summary of gene variants and clinical features of patients.

**Patient (gender)**	**Genes**	**Onset age**	**Variant**	**Inheritance, R/N**	**DS**	**Seizure types**	**ID/DD**	**Treatment**	**VEEG**	**Brain imaging**	**NGS**
1 (F)	*STXBP1*	8 d	c.1439C>T, p.Pro480Leu	*De novo*, R	OS	Tonic spasms	Severe	VPA, LEV, TPM, VB6, PB, KD, LCM	Suppression-burst	Normal	Panel
2 (M)	*STXBP1*	11 m	c.707G>A, p.Gly236Asp	*De novo*, N	EIEE	Epileptic spasms, tonic, and focal seizures	Moderate	VPA, LEV	Burst suppression and multiple focal epileptic discharges	Normal	Panel
3 (F)	*CDKL5*	2 m	c.426_429del, p.143fs	*De novo*, N	EIEE	Myoclonic, tonic, and absence seizures	Severe	VPA, TPM	Multifocal spike, sharp, and spike-slow waves	Normal	Panel
4 (F)	*CDKL5*	4 m	c.868C>T, p.Gln290Ter	*De novo*, N	WS	Epileptic spasms, absence, and focal seizures	Moderate	VPA, TPM, KD	Hypsarrhythmia and multiple focal epileptic discharges	Normal	WES
5 (M)	*PIGN*	4 m	c.2332delC, p.778fs	Paternal, N	EIEE	Epileptic spasms	Moderate	VPA, TPM, LEV, CZP, VGB	Generalised or multifocal spike, sharp, spike-slow, and polyspike-and-slow waves	Myelination delay	Panel
			c.761A>T, p.Lys254Ile	Maternal, N							
6 (M)	*PAFAH1B1*	2 m	c.530G>A, p.Trp177Ter	*De novo*, N	WS+ Pachygyria	Epileptic spasms, tonic seizures	Severe	VPA, TPM, KD	Hypsarrhythmia	Pachygyria	WES
7 (F)	*SCN1A*	6 m	c.2442C>G, p.Tyr814Ter	*De novo*, N	DS	Febrile, focal, and myoclonic seizures	Mild	VPA, LEV, KD	Background activity slow and bilateral sharp-and-slow waves	Normal	Panel
8 (M)	*SCN1A*	4 m	c.2861T>G, p.Val954Gly	*De novo*, N	DS	Febrile and focal seizures	Moderate	VPA, TPM, KD	Atypical sharp-and-slow waves	Enlarged extracerebral gap	Panel
9 (M)	*SCN1A*	3 m	c.302G>A, p.Arg101Gln	De novo, R	DS	Febrile, focal and absence seizures	Moderate	VPA, LEV, TPM, CZP	Sharp and sharp-and-slow waves, small spike waves	Normal	Panel
10 (M)	*SCN1A*	6 m	c.1624C>T, p.Arg542Ter	*De novo*, R	EIEE	Febrile and focal seizures	Severe	VPA, TPM, LTG	Background activity slow	Normal	Panel
11 (M)	*SCN1A*	4 m	c.4906C>T, p.Arg1636Ter	*De novo*, N	EIEE	Febrile, focal, and myoclonic seizures	Moderate	VPA, TPM, CZP	Sharp and sharp-and-slow waves	Normal	Panel
12 (M)	*SCN1A*	5 m	c.5170G>A, p.Ala1713Thr	*De novo*, N	DS	Febrile, focal, and myoclonic seizures	Mild	VPA, TPM, CZP	Background activity slow, spike-and-slow waves	Normal	Panel
13 (F)	*SCN1A*	6 m	c.5150T>G, p.leu1717Arg	*De novo*, N	DS	Febrile and focal seizures	Severe	VPA, TPM, CZP	Background activity slow, small spike waves	Normal	Panel
14 (M)	*SCN1A*	7 m	c.1178G>A, p.Arg393His	*De novo*, R	DS	Febrile, absence, and focal seizures	Mild	VPA, TPM, CZP	Spike-and-slow waves	Normal	Panel
15 (F)	*SCN1A*	4 m	c.2134C>T, p.Arg712Ter	*De novo*, R	DS	Febrile, absence, and myoclonic seizures	Regression	VPA, CZP, KD	Sharp and sharp-and-slow waves	Normal	WES
16 (F)	*SCN2A*	7 m	c.506A>C, p.Glu169Ala	*De novo*, N	EIEE	Focal, myoclonic, and absence seizures	Severe	TPM, VB6	Diffuse and multifocal sharp and sharp-and-slow waves	Myelin delay and cerebral atrophy	WES
17 (F)	*KCNQ2*	2 m	c.593G>A, p.Arg198Gln	*De novo*, R	WS	Epileptic spasms	Severe	VPA, LEV, TPM, ACTH	Hypsarrhythmia	Myelin delay	WES

*F, female; M, male; d, days; m, months; R, reported; N, novel; DS, diagnosis; OS, Ohtahara syndrome; EIEE, early infantile epileptic encephalopathy; WS, West syndrome; DS, Dravet syndrome; ID, intellectual disability; DD, development disability; VPA, valproate; LEV, levetiracetam; TPM, topiramate; VB6, vitamin B6; PB, phenobarbital; KD, ketogenic diet; LCM, lacosamide; CZP, clonazepam; VGB, vigabatrin; LTG, lamotrigine; ACTH, adrenocorticotropic hormone; VEEG, video-electroencephalography; NGS, next generation sequencing; WES, whole-exome sequencing*.

*STXBP1* missense variants were identified in a female and a male patient. One patient carrying c.1439C>T was diagnosed with OS and had experienced early-onset seizures since 8 days of age. The patient exhibited tonic spasms, occurring in clusters, during sleep or awakening. VEEG at seizure onset recorded burst-suppression pattern; brain MRI was unremarkable. Postnatal development was severely delayed. The patient did not respond to sodium valproate (VPA), levetiracetam (LEV), topiramate (TPM), vitamin B6 (VB6), and phenobarbital (PB). Interestingly, worsening of the seizures during the night was observed when VB6 was tapered, which improved when the initial VB6 dose was re-established. A ketogenic diet (KD) with ketone concentrations of ~3.0 mmol/L resulted in the patient being seizure-free for 10 months and was associated with cognitive improvement. After seizure relapse, lacosamide (LCM) was introduced, with good response, and the patient remained seizure-free for 6 months; developmental improvement followed. Concomitant LEV and VB6 treatment were also well-tolerated. Notably, we found that a KD was significantly associated with cognitive improvement. And the patient had no tendency to progress to WS with follow-up. The other patient, who harboured c.707G>A, was diagnosed with non-syndromic EIEE at 11 months of age, presenting with epileptic spasms, tonic seizures and focal seizures. VEEG patterns were burst suppressions, mixed with multiple focal spikes or polyspikes; brain MRI was normal. VPA and LEV were effective. After 2.5 years of treatment, the patient was free of seizures and no epileptic discharges were observed. The patient presented with moderate intellectual disability and a lisp.

*CDKL5* variants were detected in two female patients: a frameshift variant and a non-sense variant. The patient with c.426_429del had a seizure onset at 2 months and developed myoclonic, tonic and absence seizures, with no response to VPA and TPM; global developmental impairment followed. She also showed some characteristic features of Rett syndrome, such as head growth deceleration, limited hand skills, and autonomic dysfunction. Unfortunately, the patient died of frequent seizures and severe pulmonary infection at 10 months of age. The other patient, carrying c.868C>T, was diagnosed with WS at 4 months of age. She presented with moderate developmental delay and VEEG features of hypsarrhythmia and multiple focal epileptic discharges. The types included epileptic spasms, absence seizures, and focal seizures. She was treated with VPA, TPM, and KD, and being seizure free was achieved for 1 year.

*PIGN* variants were detected in one male patient. A compound heterozygous variant was identified, whereby p.778fs was inherited from his father and p.Lys254Ile from his mother. Seizure onset was noted at 4 months of age, and the patient was diagnosed with non-syndromic EIEE. The seizure type was classical epileptic spasms. VEEG showed generalised or multifocal spike, sharp, spike-slow and polyspike-and-slow waves. Brain MRI revealed myelination delay in the cerebral white matter. The patient was treated with VPA, TPM, LEV, and clonazepam (CZP), but frequent episodes could not be controlled. Later, vigabatrin (VGB) was introduced and the seizure frequency decreased markedly. The patient exhibited severe intellectual delay; he could sit alone but could not walk by himself or speak until he was 5 years old. An additional clinical feature was anomalous ongoing hypotonia.

A *PAFAH1B1* non-sense variant was detected in a male patient with intractable epilepsy and severe developmental milestone delay. The onset of seizures started at 2 months and types included epileptic spasms and tonic seizures. Brain MRI demonstrated a thickened cortex and gyral malformations. Hypsarrhythmia was identified on the VEEG record. The patient was diagnosed with WS and pachygyria, and received VPA and TPM for 4 months, but without seizure control. Then, a KD was initiated at 6 months of age, and seizure attacks in clusters decreased from more than 10 times per day to fewer than three times per day after 1 month. Nevertheless, the benefits for neurodevelopment are unclear, and frequent respiratory infections often result in second epileptic hits.

*SCN1A* variants were detected in three female patients and six male patients; Seven patients were diagnosed with DS and two with non-syndromic EIEEs. The mean onset age was 5 months. Various seizure types and developmental delays presented in the course of the disease, and VEEG showed multiple discharge abnormalities. VPA, TPM, and CZP were effective in controlling seizures in most of these patients, and seizure frequency was significantly reduced in three patients with KD treatment. After seizure remission, marked behavioural and cognitive improvements were observed. The *SCN2A* variant was detected in one female patient; the onset age was 7 months. Brain MRI revealed delayed myelin development and cerebral atrophy. High seizure frequency and seizure clustering were observed, which led to an exacerbation of early developmental delay.

A missense variant in *KCNQ2* was detected in a female patient. Her seizures started within 2 months after birth; the seizure type was epileptic spasms. Brain MRI revealed delayed myelin development and VEEG hypsarrhythmia patterns. After the diagnosis of WS, she was treated with VPA, LEV, TPM, and adrenocorticotropic hormone (ACTH), but her seizures remained uncontrolled. The patient presents severe developmental delay and suffers from recurrent pulmonary infections.

## Discussion

Because of the early age of onset and heterogeneity in the genotype and phenotype, it is difficult to identify several key phenotypic features of some of the genes responsible for EIEEs. We identified pathogenic variants in 34% of children with suspected genetic aetiology by NGS, which was similar to previous studies (8, 9). Despite progress in EIEE genetic diagnosis, there is still a limit for diagnosis using a gene panel. In the past, the gene panel was chosen prior to WES because of its cost-effectiveness, but targeted testing and inadequate coverage restricted the genetic diagnostic yield, which may miss disease-causing variants and lead a second cost of WES or whole-genome sequencing (WGS) and time. Routine use of WES or WGS can result in cost savings and higher diagnostic yields along with falling prices over time, which will lead to the discovery of new candidate genes and explanatory polygenic burdens (10). Moreover, detailed gene-specific phenotyping allowed us to seek diagnostic precision and to explore more specific approaches to treatment.

STXBP1 is predominantly expressed in the brain and plays an important role in synaptic vesicle docking and fusion (11). Variants in the *STXBP1* gene have recently been described in OS, IS, DS, and non-syndromic EIEE, termed “STXBP1 encephalopathy” (STXBP1-E). The missense variant c.1439C>T has been reported to cause a severe phenotype, and is one of the recurrent variants (12, 13). Similarly, the patient in our study presented with developmental and epileptic encephalopathy (DEE) associated with frequent seizures and early onset. The patient with c.707G>A variant also displayed DEE but with later seizure onset. The ASMs reported to be most effective are VPA and LEV (13). Both the patients responded well to VPA and LEV, but also to VB6, LCM and KD. For early-onset seizures, especially neonatal seizures, early adoption of VB6 for management might be beneficial as a promising adjunctive therapy with an unproven night “depressant” role. Although poor long-term tolerance of KD remains a barrier, we still suggest early consideration of KD for significant seizure control and improved cognition in intractable epilepsy. In fact, a previous study reported complete seizure freedom with ketone concentrations of ~4.0 mmol/L (14); in our study, we maintained it at ~3.0 mmol/L considering potential complications and tolerability at the infant stage. Recurrent episodes and active epileptiform activity can aggravate cognitive problems, and we suggest that neurologic improvement might mainly result from amelioration of epileptic activity itself through a KD. LCM is associated with a novel mechanism of selectively enhancing slow inactivation of voltage-gated sodium channels (15). A previous study showed that LCM can exacerbate tonic seizures and electrical patterns in LGS (16), and LCM tolerance with concomitant sodium channel blocking drugs remains controversial (17). A previous study also reported that infant OS patients with *STXBP1* variants may display transient responsiveness to folinic acid (18). The superiority of an ASM or a combination in STXBP1-E may require further research.

Variants of or deletions in the X-linked gene *CDKL5* have been reported to cause EEs such as X-linked infantile spasms, EIEE-2, autism spectrum disorders, and Rett-like syndrome (19), which is termed *CDKL5* deficiency disorder (CDD) (20). According to a previous study (21), the hallmarks of CDD usually include an early onset, a female predominance, intractability, typically polymorphic seizures, and severe developmental delay. Two children in this study had *CDKL5* variants that were previously unreported. The types of *CDKL5* variants may lead to differing severities of clinical phenotypes, and a deletion variant may exhibit a higher disease burden. In addition, one patient presented with a phenotype that might overlap with Rett syndrome, which was associated with MECP2 phosphorylation (22), but this feature was not detected in the WS patient. The WS patient showed a good response for a honeymoon period of 1 year without cognitive improvement. Whether the seizure-free outcome was associated with the honeymoon phase of natural disease progression in CDD or a result of ASMs is unclear (23). The severe phenotypes after an early-onset age caused by *CDKL5* variant warrants more attention.

*PIGN* is one of more than 20 genes in the phosphatidylinositol family involved in glycosylphosphatidylinositol (GPI) biosynthesis, which is associated with neurodevelopmental disorders (NDD) (24). *PIGA* and *PIGQ* variants are associated with OS, WS, and early myoclonic encephalopathy (25). Variants in *PIGN* comprised a variable phenotypes including congenital anomalies, developmental delay, hypotonia, epilepsy, gastroesophageal reflux, and progressive cerebellar atrophy (26, 27). Here, we report that a male patient diagnosed with non-syndromic EIEE carried compound heterozygous *PIGN* variants. The clinical phenotype included early onset, drug-resistant epilepsy, hypotonia, severe developmental delay, and myelination delay. The unique manifestation was epileptic spasms, and VGB is significantly effective for treating epileptic spasms without hypsarrhythmia (ESWH). Significant dysmorphic features were not observed in this patient, and previous studies also supported that congenital anomalies associated with biallelic truncating variants are not a core feature of *PIGN* variants (28, 29). Biallelic PIGN variations should be considered as one of the causes of EIEE, especially when associated with seizures, hypotonia and intellectual disability.

As previously reported, some cases of isolated lissencephaly sequences are caused by variations in the *PAFAH1B1* gene on chromosome 17p13.3 (30). Here, we reported a non-sense variant of *PAFAH1B1*, not previously reported, in a male patient. He showed an abnormal gyral pattern, drug-resistant epilepsy, and severe developmental delay. The brain MRI finding is consistent with pachygyria, a simplified convolutional pattern with few, broadened gyri and shallow sulci (31). Combined with the previous research results, non-sense variants and large deletions may be associated with more severe phenotypes than missense variants (32). We also observed the benefit of early KD implementation in the intractable condition.

*SCN1A* and *SCN2A* encode the major brain sodium channel subunits Nav1.1 and Nav1.2, respectively, which play crucial roles in the generation and propagation of neuronal firing (33). Variants in *SCN1A* and *SCN2A* have been linked to a spectrum of epilepsy phenotypes, including DS, generalised epilepsy with febrile seizures plus (GEFS+), WS and some other infantile epileptic disorders (34). The patients with *SCN1A* variants presented with early-onset seizures, drug-resistant epilepsy, developmental slowing and cognitive impairment, consistent with previous findings (35). Treatment strategies with some evidence of positive effects include VPA, clobazam, TPM, LEV, fenfluramine and bromides, or a KD (25, 36). In our study, the seizures were controlled by VPA, CZP, and KD, and we were delighted to observe improved cognitive development. In addition, the phenotypes of late-infantile onset, DEE and refractory epilepsy in the *SCN2A* variant may be associated with the loss of channel function (37). Patients with early developmental delay can benefit from genetic testing. Early and effective intervention may limit disease progression and significantly improve developmental outcomes and associated comorbidities.

*KCNQ2* variants are associated with a wide phenotypic spectrum ranging from an age-dependent, self-limiting epilepsy, to a severe DEE but also an intermediate phenotype in terms of intellectual outcomes and time to reach seizure freedom (38). In the previous study (39), the recurrent KCNQ2 variant R198Q is associated with stereotypes with refractory seizures, severely abnormal VEEG and developmental delay. In our study, the *KCNQ2* variant caused early-onset DEE and intractable seizures, and failed multidrug therapy led to a more severe developmental disorder.

Here, we explored the genetic aetiology of unexplained EIEEs and expanded the variant and phenotypic spectra. Furthermore, we elucidated the impact of the *STXBP1* variant on the protein structure. The comparison of the structures of mutant and wild-type proteins showed that the variants affected protein stability, as described further in Supplementary Figures 1, 2. The nature and position of altered amino acids might affect the protein structure at different levels, which possibly explains the differences in clinical manifestations. The major limitations of this study are the mentioned limits of gene panels and the small sample size, which does not allow for exploring statistical correlations between genes and phenotypes.

## Conclusion

NGS is a crucial tool to improve the diagnosis in EIEE and DEE and allows expanding the genotypic spectrum of this condition. Early molecular testing and detailed clinical phenotyping may lead to precise therapeutic interventions and can improve the developmental outcome. Functional studies further our understanding of novel target drugs, facilitating development in the future.

## Data Availability Statement

The datasets presented in this study can be found in online repositories. The name of the repository and accession number can be found below: National Centre for Biotechnology Information (NCBI) BioProject, https://www.ncbi.nlm.nih.gov/bioproject/, PRJNA718023.

## Ethics Statement

Written informed consent was obtained from the individual(s), and minor(s)' legal guardian/next of kin, for the publication of any potentially identifiable images or data included in this article.

## Author Contributions

JC and XLiu: conceptualisation and writing. XLiu and QS: analysis. XW, XY, and ZC: data collection and confirmation. JC, GZ, HG, and XLu: project administration and supervision. All authors contributed to the article and approved the submitted version.

## Conflict of Interest

The authors declare that the research was conducted in the absence of any commercial or financial relationships that could be construed as a potential conflict of interest.
