# Supplementary material for: Gene and Phenotype Expansion of Unexplained Early Infantile Epileptic Encephalopathy
Source: Front Neurol. 2021 Jun 7;12:633637. doi: 10.3389/fneur.2021.633637 (PMC8215605; doi:10.3389/fneur.2021.633637)
Supplement: Supplementary file 1 [file Data_Sheet_1.DOCX]

**Figure 1**


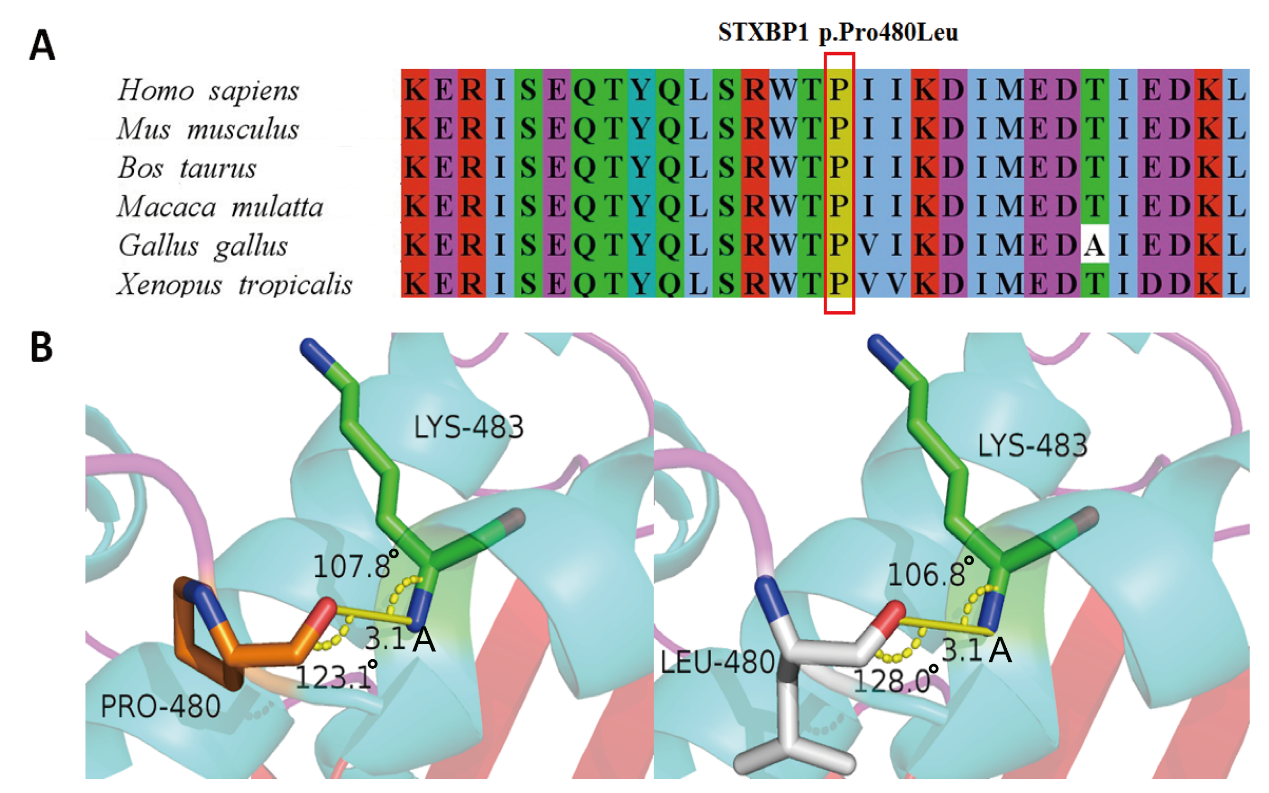


**Figure 2**


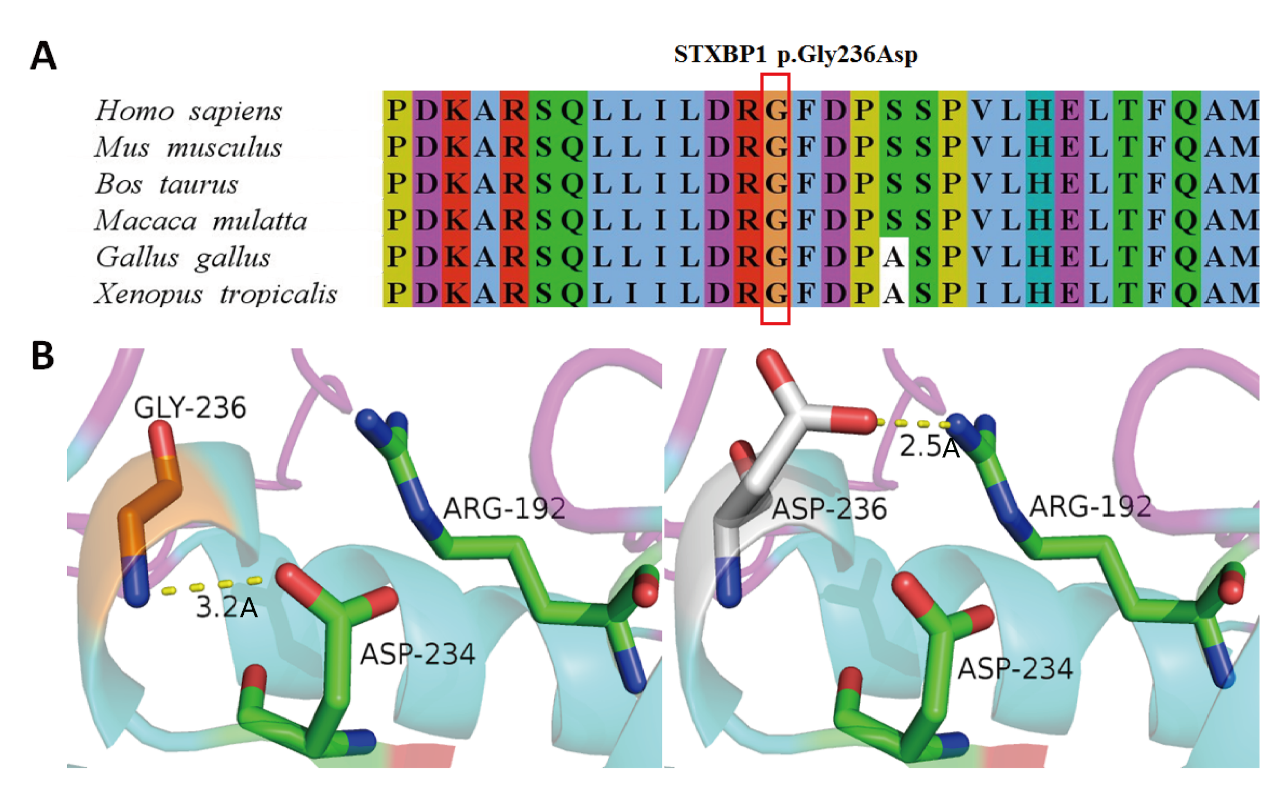


**Figure 1, 2. (A):** The ClustalX comparison of amino acid sequences for the variant neighbor segment shows all conservation across different species respectively.

**(B):** Positions of the missense variants (Pro480Leu, Gly236Asp) in the STXBP1 crystal structure are shown as a stereo representation. Compared with hydrogen bonds (yellow solid lines) of residues Pro480 and Lys483, the bond lengths are the same, but there is a slight change of bond angle between Leu480 and Lys483. Pyrrolidine ring lack of PRO might devastate the flexibility of peptide chain and thermal stability of protein when PRO is mutated to Leu. Gly236 forms a hydrogen bond (yellow broken line) with the residue Asp234, but mutated amino acid (Asp236) causes a new remodeling binding to Arg192.
